# Uterine artery pulsatility index: a predictor of methotrexate resistance in gestational trophoblastic neoplasia

**DOI:** 10.1038/bjc.2012.65

**Published:** 2012-02-28

**Authors:** R Agarwal, V Harding, D Short, R A Fisher, N J Sebire, R Harvey, D Patel, P M Savage, A K P Lim, M J Seckl

**Affiliations:** 1 Department of Medical Oncology, Charing Cross Gestational Trophoblastic Disease Centre, Charing Cross Hospital, Imperial College Healthcare NHS Trust, Fulham Palace Road, London W6 8RF, UK

**Keywords:** low-risk GTN, UAPI, drug resistance, angiogenesis

## Abstract

**Background:**

Neo-angiogenesis is a hallmark of cancer. The aim of this study was to test the hypothesis, in a prospective patient cohort, that in low-risk gestational trophoblastic neoplasia (LR-GTN) the uterine artery pulsatility index (UAPI), a measure of tumour vascularity, can predict resistance to methotrexate chemotherapy (MTX-R).

**Methods:**

286 LR-GTN patients (Charing Cross Hospital (CXH) score 0–8, or FIGO score 0–6) were treated with methotrexate between January 2008 and June 2011 at CXH. During staging investigations, patients underwent a Doppler ultrasound to assess the UAPI.

**Results:**

239 patients were assessable for both UAPI and MTX-R. The median UAPI was lower (higher vascularity) in MTX-R compared with MTX-sensitive patients (0.8 *vs* 1.4, *P*<0.0001). In multivariate logistic regression, UAPI⩽1 predicted MTX-R, independent of both CXH and FIGO scores. The risk of MTX-R in patients with a FIGO score of 6 and UAPI⩽1 was 100% *vs* 20% in patients with UAPI>1 (*χ*^2^ *P*<0.0001).

**Conclusion:**

UAPI represents an independently validated clinically useful predictor of MTX-R in LR-GTN. Further, consideration of whether to incorporate UAPI into the FIGO scoring system is now warranted so that patients with a score of 6 and a UAPI ⩽1 might be upstaged and offered combination chemotherapy rather than MTX.

Gestational trophoblastic neoplasia (GTN) is one of the few malignancies that can be cured by cytotoxic chemotherapy, even when widespread disease is present. The GTN encompasses a spectrum of histological entities including invasive mole, choriocarcinoma, placental site trophoblastic tumour and the extremely rare epitheloid trophoblastic tumour (Shih Ie, 2007; Seckl *et al*, 2010). Commonly these arise from molar pregnancies, although they can arise from any type of pregnancy including term deliveries, ectopic pregnancies and miscarriages. Molar pregnancies are classified as either partial (PHM) or complete hydatidiform moles (CHM) (Kajii and Ohama, 1977; Szulman and Surti, 1978a, 1978b). In UK, the incidence of CHM is about 1 out of 1000 pregnancies with PHM more common (3 out of 1000). The risk of persistent GTN is about 15% with CHM and 0.5–1% with PHM (Smith, 2003). In UK, all patients diagnosed with molar pregnancies are registered in one of the three centres (Dundee for Scotland, Sheffield for Northern England and London for the rest of the UK) to enable centralised pathological review and surveillance using serial human chorionic gonadotrophin (hCG) measurements according to an established protocol (Seckl *et al*, 2010).

If a rise or plateau in hCG is detected during surveillance, this indicates the likely onset of malignant change requiring chemotherapy. Additional criteria for commencing chemotherapy in these patients, which include a histological diagnosis of choriocarcinoma and the presence of metastases to the brain and gastro-intestinal tract, are shown in Supplementary Table 1.

The chemotherapy used is based on the FIGO prognostic scoring system, and has been in use in its present form since 2002 (Supplementary Table 2). It has super-ceded previous scoring systems, including the Charing Cross Hospital (CXH) scoring system developed by Bagshawe (Bagshawe, 1976). A FIGO score of 7 (CXH score of 9) or more denotes disease that is at high risk of failure to single-agent chemotherapy, and these patients are treated from the outset with etoposide, methotrexate and Dactinomycin alternating weekly with cyclophosphamide and vincristine (EMA/CO) (Bower *et al*, 1997; Aghajanian, 2011). A FIGO score of ⩽6 (CXH score 8) denotes disease that has a ‘low risk’ of developing resistance to single-agent therapy with methotrexate or Dactinomycin (McNeish *et al*, 2002; Aghajanian, 2011). However, about 30% of these low-risk patients become resistant to single-agent chemotherapy and require combination chemotherapy most commonly in the form of EMA/CO (Table 1) (McNeish *et al*, 2002; Seckl *et al*, 2010; Aghajanian, 2011). The majority of the low-risk patients are salvaged and the overall survival in low-risk patients is now virtually 100% (McNeish *et al*, 2002). However, switching chemotherapy following development of resistance to single-agent methotrexate (MTX-R) prolongs the overall duration of chemotherapy and can cause considerable psychological distress to patients. Consequently, there is a need to further refine the scoring system so that we can more accurately identify the 30% of patients destined to become resistant to single-agent therapy.

Neo-angiogenesis, the formation of new blood vessels, is a critical step in tumourogenesis (Hanahan and Weinberg, 2011). Neo-angiogenesis is associated with increased tumour growth, acquisition of metastatic potential, drug resistance and poor prognosis in a number of solid tumours such as breast, lung and ovarian cancer (Weidner *et al*, 1992; Weidner, 1993; Fontanini *et al*, 1995; Hollingsworth *et al*, 1995; Siddiqui *et al*, 2010). Histological asssessment of microvessel density (MVD) with CD34 immunostaining is used to assess angiogenesis in these tumours (Weidner, 1993). In contrast, MVD assessment is not possible in GTN as biopsy is frequently contraindicated because of the risk of precipitating life-threatening haemorrhage in this highly vascular disease. Instead, the diagnosis is usually made on the basis of rising hCG levels post-molar pregnancy. We previously proposed the use of Doppler ultrasonography as a non-invasive alternative to assess tumour vascularity in GTN, using the uterine artery pulsatility index (UAPI) (Agarwal *et al*, 2002). The UAPI is inversely proportional to tumour vascularity, and a low UAPI is indicative of increased arteriovenous shunting, a feature of the abnormal neo-angiogeneisis characteristic of tumours. In that study of 164 patients with GTN, a UAPI of ⩽1 was shown to be an independent predictor of MTX-R and in combination with the CXH scoring system improve prediction of MTX-R. In particular, the risk of MTX-R in patients with medium-risk CXH scores of 6–8 with a UAPI of ⩽1 was increased to 72.7% from a baseline risk of 56.3% in this group. Our findings suggested that UAPI might be a useful additional variable to incorporate into the prognostic scoring systems to help refine which patients might be treated with EMA-CO chemotherapy upfront (Agarwal *et al*, 2002).

The aim of this study was to test this hypothesis in an independent patient cohort, and demonstrate that the UAPI should be used in addition to the FIGO score for patient stratification for chemotherapy in GTN.

## Methods

### Patients

All patients (*n*=286) treated upfront with single agent MTX-R for GTN between January 2008 and June 2011 were identified from the Charing Cross Hospital Trophoblastic Disease Database (CXH-TDD). We reviewed the patient characteristics including age, hCG level, number and location of metastatic disease at presentation, histological diagnosis and CXH and FIGO scores. Those patients that were switched to a second-line chemotherapy (either single agent Dactinomycin or EMA-CO) were considered resistant to methotrexate if the change was the result of a plateaued or rising hCG. Patients who were switched due to drug side-effects (serositis or allergy) were not considered to be resistant. In the CXH prognostic scoring system, a patient with a score between 0 and 5 is at low risk of MTX-R. Patients scoring 6 to 8 and >8 are at medium- and high-risk of MTX-R, respectively. Only patients with CXH scores <9 or FIGO scores <7 at baseline are treated with single-agent methotrexate (18) and were therefore included in this study; high-risk patients were excluded. The details of individual prognostic factors, CXH and FIGO prognostic scores, and treatment details were obtained for all patients in the study from the CXH-TDD.

Patients underwent a single pelvic ultrasonographic examination before chemotherapy. The results of Doppler assessments were obtained from the original US reports. These reports were missing, or inadequately documented in 47 patients. Because our intention in this study was to examine the relationship between tumour vascularity measured by Doppler US and MTX-R, these patients were excluded from subsequent analysis.

### Uterine artery pulsatility index

The total uterine volume and the UAPI were measured as described previously. Doppler US took an average of 15–20 min to complete per patient. Doppler assessments were performed using an Aplio XG ultrasound scanner (Toshiba Medical Systems, Nasu, Japan) with a 2–5 MHz curvilinear array probe. Uterine volume was calculated using the prolate ellipsoid formula: uterine volume (cm^3^)=*L* (cm) × *AP* (cm) × *W* (cm) × 0.523, where *L* is length, *AP* is maximum antero-posterior diameter, and *W* is maximum width (1 cm^3^=1 ml).

UAPI was chosen to assess blood flow in this study, because it is independent of the angle of insonation, and this angle cannot reliably be estimated for uterine arteries because of their small diameter and tortuosity. The UAPI is given by the formula: UAPI=(*A*−*B*)/mean, where *A*, *B*, and the mean are the maximum, minimum, and time averaged Doppler frequency shift of the ultrasound beam after reflection from the moving column of blood in the uterine artery. The UAPI was calculated by averaging the values from a minimum of three cardiac cycles using the scanner software. The UAPI reflects the impedance to flow distal to the point of sampling; an increase in impedance will result in an increase in the UAPI and *vice versa*. Using power Doppler, the uterine arteries were located before spectral Doppler analysis, and both uterine arteries were examined. The lowest UAPI from either uterine artery was used for analysis, because it is a reflection of the maximal deviation from the normal impedance.

### Chemotherapy and response evaluation

Patients were initially treated with fortnightly cycles of 50 mg of methotrexate i.m. on days 1, 3, 5 and 7, with 15 mg of oral folinic acid rescue on days 2, 4, 6 and 8 (the MTX regimen) (McNeish *et al*, 2002). Response to chemotherapy was monitored by twice weekly serum hCG measurements. Methotrexate chemotherapy was defined by a plateau or a rise in two consecutive hCG concentrations (McNeish *et al*, 2002). Patients with MTX-R were changed to Dactinomycin 0.5 mg i.v., daily for 5 days every 2 weeks, if their hCG concentration at resistance was ⩽300 IU l^–1^. Otherwise, patients were treated with EMA/CO given intravenously as a weekly alternating schedule (Bower *et al*, 1997; McNeish *et al*, 2002). Treatment was continued in all patients for 6 weeks beyond the fall of the hCG to normal (⩽4 IU l^–1^).

### Statistical analysis

SPSS V17.0. (SPSS, Chicago, IL, USA) was used for statistical analyses. Univariate analyses were performed using the Mann–Whitney *U* test or *χ*^2^ test. UAPI was subdivided into high- and low-risk categories for MTX-R using a cut of ⩽1 and >1, defined in our previous study based on ROC curve analysis (Agarwal *et al*, 2002). These UAPI subgroups were used in multivariate analyses to predict MTX-R, using binary logistic regression with forward stepwise selection. The CXH or FIGO prognostic scores were included as co-variates. In all models, the prognostic score was always included and the additional utility of UAPI assessed. The CXH score was dichotomised into low- (0–5) and medium- (6–8) risk groups as previously published. The equivalent FIGO categories used were 0–5 *vs* 6, and 0–4 *vs* 5–6 to assess the impact of UAPI using current FIGO scores.

With 239 patients, and using the pre-defined UAPI cutoff of 1, which was the median in the previous study, and risk of MTX-R of 35% and 65% for patients with an UAPI>1 and ⩽1, respectively (Agarwal *et al*, 2002), the current study had a power of 99.8% to detect a difference in risk of MTX-R with a two-sided alpha of 5%.

## Results

**Figure 1:**
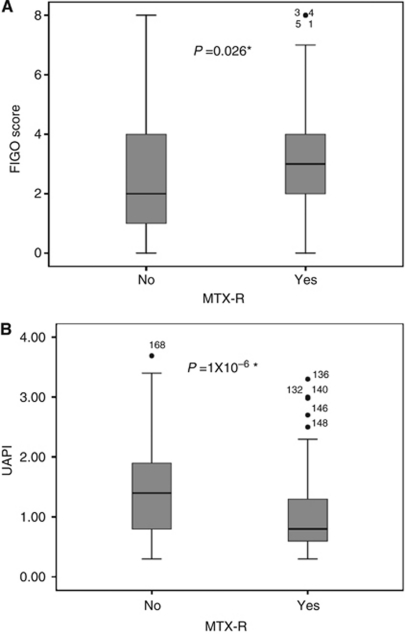
Box-plots of FIGO score (**A**) and UAPI (**B**) by resistance to MTX-R. ^*^Mann–Whitney *U* test, Resistant=YES (*n*=113), Sensitive=NO (*n*=126).

Between January 2008 and June 2011, 286 patients with low-risk GTN were treated with single agent intra-muscular methotrexate. 239 patients with quantitative UAPI results available from baseline pre-chemotherapy assessments were eligible and all were chemo-naive at presentation. Three had histologically confirmed choriocarcinomas.The median (range) age of patients was 33 years (15–55 years), serum hCG 11 865 IU ml^–1^ (12–230 836 IU ml^–1^), CXH score 3 (0–8) and FIGO score 3 (0–6; Table 1). The median UAPI was 1 (range 0.3–3.69; Figure 1). In keeping with a previous report demonstrating a rise in MTX-R with the FIGO scoring system (El-Helw *et al*, 2009), 45% of patients developed MTX-R and switched to either dactinomycin (*n*=58) or EMA/CO (*n*=55) chemotherapy (Table 1).

On univariate analysis, the baseline CXH (Mann–Whitney *U* test, *P*=0.026) and FIGO (*P*=0.039) scores were associated with MTX-R (Table 1; Figure 1). Of the components of the CXH and FIGO scores, only high hCG levels were significantly associated with MTX-R (*P*=0.011, Table 1). In contrast, baseline UAPI was significantly lower in patients who subsequently developed MTX-R compared with methotrexate-sensitive patients (Mann–Whitney *U* test, *P*=1x10^−6^) (Figure 1). Using a previously defined cutoff of 1 for UAPI, UAPI⩽1 *vs* >1 was predictive of MTX-R with rates of 64.6% *vs* 35.4%, respectively (*χ*^2^, *P*=4x10^−5^; Table 2). The ROC area under the curves were 0.68 (*P*<0.0001) and 0.58 (*P*=0.03) for prediction of MTX-R using UAPI and FIGO scores, respectively (Supplementary Figure 1).

In multivariate analyses, UAPI (⩽1 *vs* >1) was an independent predictor of MTX-R with odds ratios (ORs) of 2.82 (*P*<0.0001) or 2.82 (*P*<0.0001 relative to the CXH score (model A) or FIGO score (model C) considered as continuous variables, respectively (Table 3). With the CXH score dichotomised into low- (0–5) and medium-risk (6–8) groups as described previously (model B), UAPI remained a significant independent predictor of MTX-R (OR 2.98, *P*<0.0001; Table 3). The absolute risk of MTX-R was 100% in patients with CXH scores of 6–8 and UAPI ⩽1, compared with a risk of 33% in patients with a UAPI>1 (*χ*^2^, *P*<0.0001; Table 4).

The current FIGO system was not designed to divide patients into low and medium risks. Comparing the CXH and FIGO scores among patients, the equivalent FIGO groups used for low and medium risk were 0–4 and 5–6, respectively (data not shown). Using these FIGO score categories (model D), UAPI remained an independent predictor of MTX-R (OR 2.9, *P*<0.0001; Table 3). The addition of UAPI to the FIGO score significantly improved prediction of MTX-R, with the absolute risk of MTX-R of 81% in patients with a FIGO score of 5–6 and UAPI ⩽1, compared with a risk of 13% in patients with a UAPI>1 (*χ*^2^, *P*<0.0001; Table 4). With FIGO low- and medium-risk groups defined using scores of 0–5 and 6 (model E), respectively, UAPI remained a significant predictor (OR 3.0, *P*<0.0001; Table 3) the risk of MTX_R in patients with a UAPI⩽1 and FIGO score of 6 was 100%, compared with 20% in patients with a UAPI>1 (*χ*^2^, *P*<0.0001; Table 4).

## Discussion

This study demonstrates that increased uterine blood flow, assessed using the UAPI on Doppler sonography, is associated with an increased risk of methotrexate resistance. These results validate our previous study, and establish UAPI as an independent predictor of methotrexate resistance. So how can we use the new information regarding UAPI to select only those patients who will definitely fail single-drug therapy and really need combination agent therapy?

The FIGO scoring system for stratification of patients with GTN to single agent *vs* multi-agent first-line chemotherapy is an established method for treatment selection(Aghajanian, 2011). However, despite its utility over a third of patients incorrectly receive single-agent MTX-R using this approach, so improvements to this scoring system are still required (McNeish *et al*, 2002). In our previous study, we found that a UAPI⩽1 and CXH score 5–8 was associted with a 73% risk of MTX-R compared with a 56% risk in patients with a UAPI>1, with an OR of 2.7 (Agarwal *et al*, 2002). In the current study, we confirmed this increase in risk in patients, and equivalent odds rato (OR 2.8), with respect to the CXH score. The impact of UAPI was maintained with the current FIGO scoring system, including the quantitative risk of MTX-R (OR 2.8–3). In the FIGO system we would recommend using a score of 6 and a UAPI⩽1, as the threshold for upfront multi-agent chemotherapy rather than single-agent methotrexate based on a MTX-R risk of 100% in these patients (Table 4).

This could be achieved either through the use of UAPI alongside the FIGO score, or its quantitative incorporation into the scoring system. On the basis of the ORs for UAPI in multivariate analysis we would suggest the addition of 1 point for a UAPI⩽1. Indeed, a modified FIGO score with 1 point added to the original FIGO score performed better than the original score at predicting MTX-R, in multivariate analyses (data not shown). Pragmatically, this would have the desired effect of increasing the score of patients currently with 6 points to 7, and therefore a change in their preferred first-line therapy to EMA/CO rather than methotrexate.

Doppler USS is a quick and cheap method of assessing GTN vascularity, with global availability, and can be easily incorporated into existing baseline USS assessments of patients before chemotherapy. The coefficient of variation of UAPI is <10%. The remarkable similarity in median UAPI of 1 and associated risk between the current and previous study also attests to the relative independence of the impact of UAPI with respect to ultrasound equipment used as we have moved through several generations of scanners, and as such should be feasible for almost all centres treating GTN to perform during staging pelvic ultrasound. We would however recommend a high-end ultrasound scanner with a good Doppler sensitivity and profile. The UAPI measurements and Doppler ultrasound should also be carried out by sonographers or sonologists with experience of assessing molar pregnancies.

The current study was performed in a single institution (Charing Cross Hospital) patient cohort, and as such one limitation of our study is the potential for selection and ascertainment bias common to unblinded non-randomised studies. The patients, however, represent a consecutive series of low-risk gestational trophoblastic neoplasia (LR-GTN), and CXH is one of the two national referral centres, and as such our results are unlikely to be subject to referral/selection bias compared with centres without national registration and treatment policies. The effect of ascertainment bias on our results, are therefore likely to be minimal as the end-point of MTX-R was based on quantitative serial hCG measurements, assessed independently of the UAPI. Low-risk patients with an hCG⩾400 000 IU l^–1^ will have started first-line EMA-CO based on our previous results showing the high risk of methotrexate resistance in these patients, and were excluded. The effect of this is that our reported risk of MTX-R in patients with a UAPI⩽1 and high FIGO score (5–6), is likely to be an underestimate (McGrath *et al*, 2010). Another potential limitation is the modest size of the current study with 239 patients. However, in combination with the previous study of 164 patients, giving a total of 403 patients, this exceeds the study size of 317 on which the entire CXH/FIGO scoring system was initially derived (Bagshawe, 1976; Agarwal *et al*, 2002). The combination of UAPI⩽1 and CXH score 5–8/FIGO score 6, improves predictive accuracy by 9%, but identifies only a small but still significant proportion (4.5%) of all patients who develop MTX-R. Confirmatory prospective randomised trials of UAPI are therefore unlikely to be feasible, although we are exploring this possibility within a planned GOG study.

The incorporation of UAPI as a marker of tumour vascularity and angiogenesis provides a novel additional biological facet to the FIGO score. The FIGO/CXH scores are essentially based on measures of total tumour burden (e.g., hCG, largest mass and number of metastases) and metastasis (e.g., number of metastases and sites of metastases) (Bagshawe, 1976; Aghajanian, 2011). This is reflected in the strong correlation between serum hCG levels and FIGO/CXH scores (Spearman correlation coefficients 0.63 and 0.70, *P*<0.0001). Tumour neo-angiogenesis is a hallmark of solid tumours and critical for tumour growth and metastasis (Hanahan and Weinberg, 2011). Angiogenesis in some cancers can directly enhance tumour aggressiveness and chemoresistance by activating growth, survival, proliferation and anti-apoptotic signalling pathways through angiogeneic moieties such as basic fibroblast growth factor, vascular endothelial growth factor and platelet-derived growth factors (Yang *et al*, 2009; Linderholm *et al*, 2010; Carmo *et al*, 2011).

Interestingly, hCG can also act as an angiogenic factor in placental trophoblastic tissues. However, the correlation between UAPI and serum hCG levels was limited in the current study (Spearman correlation coefficients −0.275, *P*<0.0001), and suggests that angiogenic factors other than hCG likely drive tumour vascularity in GTN (Zygmunt *et al*, 2002; Berndt *et al*, 2006; Cole, 2010). In an ongoing study, we are assessing a panel of known circulating angiogenesis factors and correlating these with the UAPI to establish drivers of angiogenesis, and with MTX-R as alternatives to UAPI. Interestingly, in a recent study Shih Ie (2011) has shown that choriocarcinomas despite their apparent vascularity, lack endothelial-lined intra-tumoural blood vessels, and instead exhibit vascular mimicry. Consequently, it is plausable that the biological drivers of this may be distinct from classical angiogenic factors (Shih Ie, 2011). In the current study, only three patients had choriocarcinomas. Exclusion of these patients did not significantly alter our results, and due to the small numbers we were unable to assess the impact of vascular mimicry on UAPI in choriocarcinomas.

In conclusion, this study validates the utility of UAPI as a non-invasive marker of tumour vascularity, and combined with the previous study, confirms in a total cohort of over 400 patients, the clinical utility of UAPI for MTX-R prediction in LR-GTN, independent of CXH/FIGO scores. These results suggest that upfront EMA/CO chemotherapy could be considered for patients with GTN with CXH scores 6–8 or FIGO score 6, and a UAPI ⩽1, respectively. In the future, this might be achieved by adding one point for a UAPI⩽1 to the FIGO score.

## Figures and Tables

**Table 1:** Patient characteristics

		**MTX-R**				
	**All**	**Yes**		**No**		***χ* ^2^**
	***n*=239**	***n*=113**	**%**	***n*=126**	**%**	***P***
*Age (years)*						
Median	33	32	15–52	33	15–55	0.379^a^
						
*Age score*						
0	189	94	83	95	75	0.139
1	50	19	17	31	25	
						
*Pregnancy score*						
0	226	106	94	120	95	0.835
1	8	4	4	4	3	
2	5	3	3	2	2	
						
*Interval score*						
0	222	105	93	117	93	0.996
1	15	7	6	8	6	
2	2	1	1	1	1	
						
*hCG score*						
0	46	14	12	32	25	0.011
1	67	29	26	38	30	
2	120	65	58	55	44	
4	6	5	4	1	1	
						
*No. of metastases score*						
0	214	100	88	114	90	0.617
1	25	13	12	12	10	
						
*Site of metastasis score*						
0	239	113	100	126	100	NA
						
*Large mass score*						
0	112	48	42	64	51	0.256
1	74	35	31	39	31	
2	53	30	27	23	18	
						
*FIGO score*						
Median	3	3	0–6	2	0–6	0.026^a^
0–4	213	97	86	116	92	0.234
5–6	24	14	12	10	8	
0–3	169	76	67	93	74	0.364
4–6	68	35	31	33	26	
						
*CXH score*						
Median	3	3	0–8	3	0–8	0.039^a^
0–5	226	104	92	122	97	0.103
6–8	13	9	8	4	3	

Abbreviations: CXH=Charing Cross Hospital; FIGO=International Federation Gynecology and Obstetrics; hCG=human chorionic gonadotrophin; MTX-R=methotrexate chemotherapy.

a Mann–Whitney *U* test.

**Table 2:** UAPI and resistance to MTX-R

		**UAPI**				
	**All**	**⩽1**		**>1**		***χ* ^2^**
**MTX-R**	***n*=239**	***n*=121**	**%**	***n*=118**	**%**	***P***
NO	126	48	40	78	66	4 × 10^−5^
YES	113	73	60	40	34	

Abbreviations: MTX-R=methotrexate chemotherapy; UAPI=uterine artery pulsatility index.

**Table 3:** Multivariate analysis of independence of UAPI relative to CXH and FIGO scores (CXH or FIGO scores were included in all models and UAPI included if significant by forward selection)

							**95% CI for EXP(B)**		
**Model**	**Variables**	**Categories**	**B**	**Wald**	**df**	**Exp(B)**	**Lower**	**Upper**	***P***
A	CXH score	Continuous	0.142	3.223	1	1.152	0.987	1.346	0.073
	UAPI	>1 *vs* ⩽1^a^	1.037	14.593	1	2.821	1.657	4.804	<0.0001
B	CXH score	Low (0–5) *vs* medium risk (6–8)^a^	1.002	2.486	1	2.725	0.784	9.476	0.115
	UAPI	>1 *vs* ⩽1^a^	1.093	16.301	1	2.983	1.755	5.072	<0.0001
C	FIGO score	Continuous	0.151	2.847	1	1.163	0.976	1.385	0.092
	UAPI	>1 *vs* ⩽1^a^	1.038	14.377	1	2.822	1.651	4.825	<0.0001
D	FIGO score	Low (0–4) *vs* medium risk (5–6)^a^	0.350	0.599	1	1.419	0.585	3.443	0.439
	UAPI	>1 *vs* ⩽1^a^	1.079	15.756	1	2.941	1.726	5.009	<0.0001
E	FIGO score	Low (0–5) *vs* medium risk (6)^a^	0.606	0.786	1	1.833	0.480	6.998	0.375
	UAPI	>1 *vs* ⩽1^a^	1.103	16.553	1	3.014	1.771	5.128	<0.0001

Abbreviations: CI=confidence interval; CXH=Charing Cross Hospital; UAPI=uterine artery pulsatility index.

a Reference category.

**Table 4:** Risk of resistance to MTX-R by CXH or FIGO score and UAPI

	**UAPI**								
	**>1**				**⩽1**				***χ* ^2^**
**MTX-R**	***n***	**%**	***n***	**%**	***n***	**%**	***n***	**%**	***P***
	CXH score (0–5)		CXH score (6–8)		CXH score (0–5)		CXH score (6–8)		
No	74	66	4	67	48	42	0	0	<0.0001
Yes	38	34	2	33	66	58	7	100	
	FIGO score (0–4)		FIGO score (5–6)		FIGO score (0–4)		FIGO score (5–6)		
No	71	65	7	88	45	43	3	19	<0.0001
Yes	38	35	1	13	59	57	13	81	
	FIGO score (0–5)		FIGO score (6)		FIGO score (0–5)		FIGO score (6)		
No	74	66	4	80	48	42	0	0	<0.0001
Yes	38	34	1	20	67	58	5	100	

Abbreviations: CXH=Charing Cross Hospital; MTX-R=methotrexate chemotherapy; UAPI=uterine artery pulsatility index.
