# Supplementary material for: Uterine artery pulsatility index: a predictor of methotrexate resistance in gestational trophoblastic neoplasia
Source: Br J Cancer. 2012 Feb 28;106(6):1089–94. doi: 10.1038/bjc.2012.65 (PMC3304432; doi:10.1038/bjc.2012.65)
Supplement: Supplementary Information [file bjc201265x1.ppt]

## Slide 1
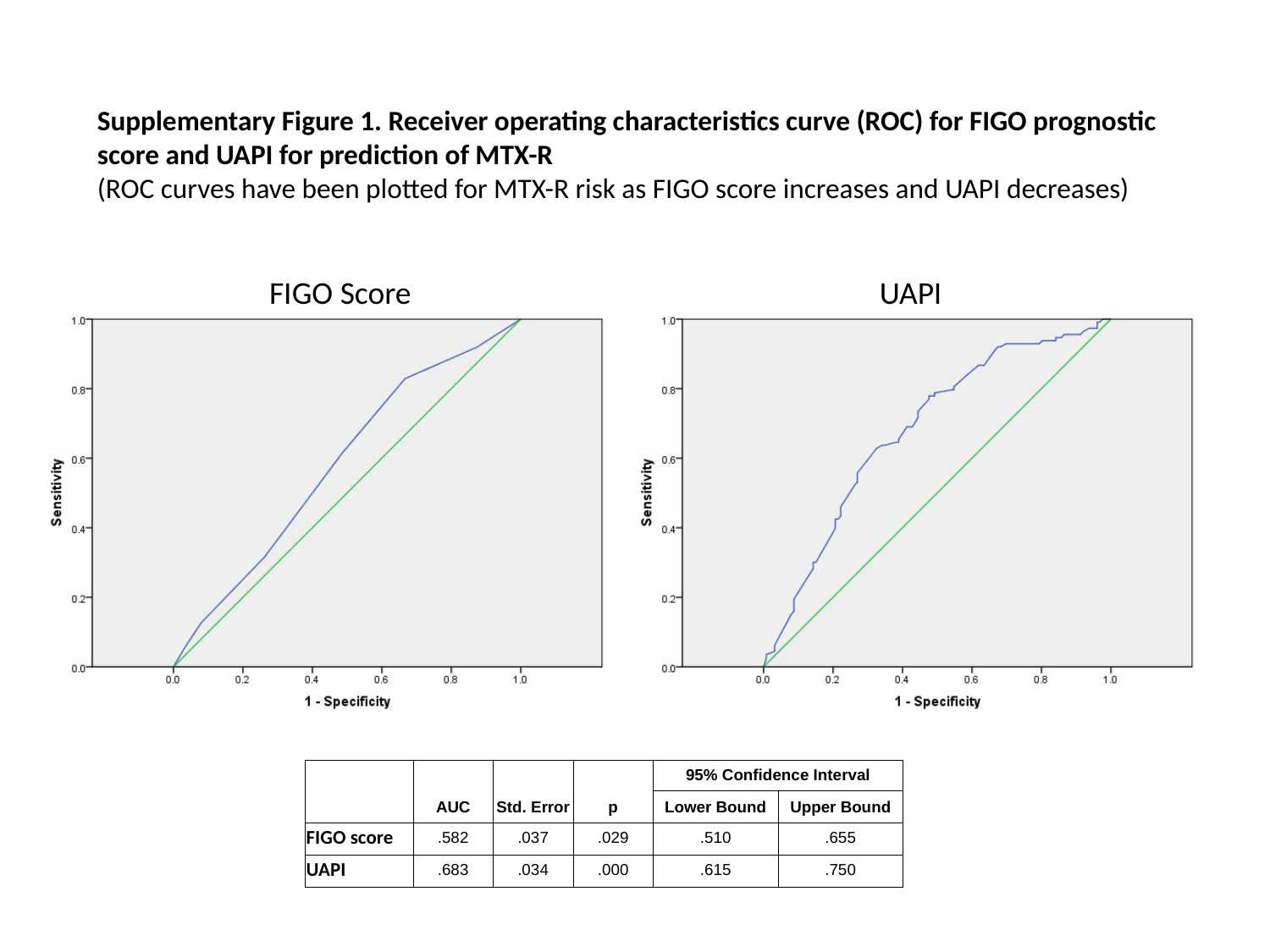

Supplementary Figure 1. Receiver operating characteristics curve (ROC) for FIGO prognostic score and UAPI for prediction of MTX-R
(ROC curves have been plotted for MTX-R risk as FIGO score increases and UAPI decreases)
FIGO Score
UAPI
| | AUC | Std. Error | p | 95% Confidence Interval | |
| --- | --- | --- | --- | --- | --- |
| | | | | Lower Bound | Upper Bound |
| FIGO score | .582 | .037 | .029 | .510 | .655 |
| UAPI | .683 | .034 | .000 | .615 | .750 |

## Slide 2
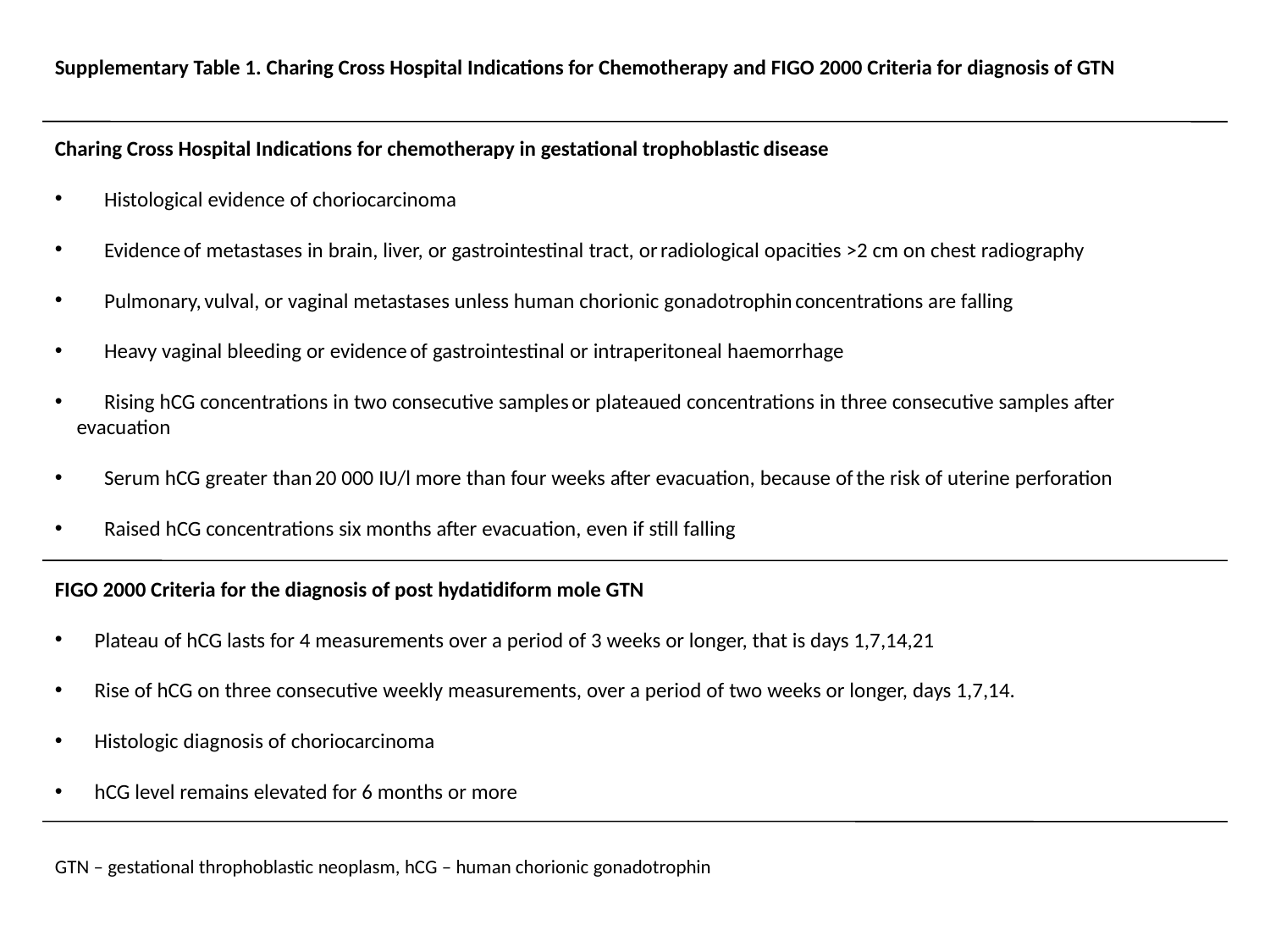

Supplementary Table 1. Charing Cross Hospital Indications for Chemotherapy and FIGO 2000 Criteria for diagnosis of GTN
Charing Cross Hospital Indications for chemotherapy in gestational trophoblastic disease
 Histological evidence of choriocarcinoma
 Evidence of metastases in brain, liver, or gastrointestinal tract, or radiological opacities >2 cm on chest radiography
 Pulmonary, vulval, or vaginal metastases unless human chorionic gonadotrophin concentrations are falling
 Heavy vaginal bleeding or evidence of gastrointestinal or intraperitoneal haemorrhage
 Rising hCG concentrations in two consecutive samples or plateaued concentrations in three consecutive samples after
 evacuation
 Serum hCG greater than 20 000 IU/l more than four weeks after evacuation, because of the risk of uterine perforation
 Raised hCG concentrations six months after evacuation, even if still falling
FIGO 2000 Criteria for the diagnosis of post hydatidiform mole GTN
 Plateau of hCG lasts for 4 measurements over a period of 3 weeks or longer, that is days 1,7,14,21
 Rise of hCG on three consecutive weekly measurements, over a period of two weeks or longer, days 1,7,14.
 Histologic diagnosis of choriocarcinoma
 hCG level remains elevated for 6 months or more
GTN – gestational throphoblastic neoplasm, hCG – human chorionic gonadotrophin

## Slide 3
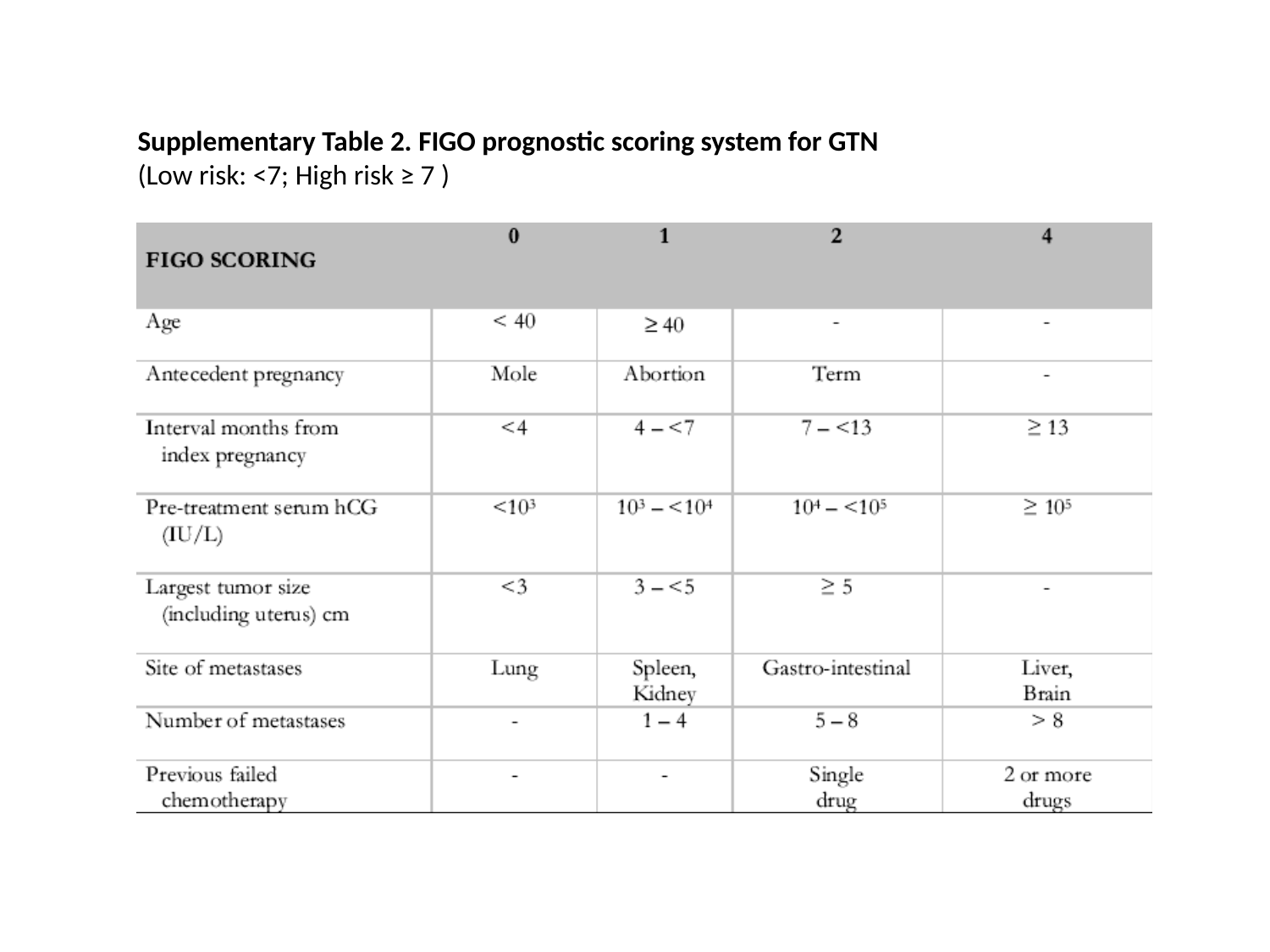

Supplementary Table 2. FIGO prognostic scoring system for GTN
(Low risk: <7; High risk ≥ 7 )
